# The impacts of mining on soil pollution with metal(loid)s in resource-rich Mongolia

**DOI:** 10.1038/s41598-023-29370-w

**Published:** 2023-02-16

**Authors:** Václav Pecina, David Juřička, Josef Hedbávný, Martin Klimánek, Jindřich Kynický, Martin Brtnický, Renata Komendová

**Affiliations:** 1 Institute of Chemistry and Technology of Environmental Protection, Faculty of Chemistry, Brno University of Technology, Purkyňova 118, 612 00 Brno, Czech Republic; 2 Department of Geology and Soil Science, Faculty of Forestry and Wood Technology, Mendel University in Brno, Zemědělská 3, 613 00 Brno, Czech Republic; 3 Department of Chemistry and Biochemistry, Faculty of Agrisciences, Mendel University in Brno, Zemědělská 1, 613 00 Brno, Czech Republic; 4 Department of Forest Management and Applied Geoinformatics, Faculty of Forestry and Wood Technology, Mendel University in Brno, Zemědělská 3, 613 00 Brno, Czech Republic; 5 BIC Brno, Technology Innovation Transfer Chamber, Purkyňova 125, 612 00 Brno, Czech Republic; 6 Department of Agrochemistry, Soil Science, Microbiology and Plant Nutrition, Faculty of AgriSciences, Mendel University in Brno, Zemědělská 1, 613 00 Brno, Czech Republic

**Keywords:** Environmental sciences, Environmental impact

## Abstract

As Mongolia is considered one of the most resource extraction-dependent countries globally, significant mining-related environmental and human health risks are expected. The aim of this study was to (I) assess the impacts of mining on soil pollution with metals in Mongolia's key coal mining towns (Baganuur, Nalaikh and Sharyn Gol) and (II) review the current knowledge on soil pollution with metal(loid)s and related health risks in Mongolia. The results showed predominantly low soil contents of Cd, Cu, Pb and Zn and a related absence of severe pollution and potential health risk in the coal mining towns. Urban design, rather than the presence of mines, controlled the pollution distribution. Despite the methodological shortcomings of several studies on soil pollution in Mongolia, their results suggest a similarly low threat in the three largest cities (Ulaanbaatar, Darkhan, Erdenet) and several mining areas. While the generally highlighted risk of As seems like an artificially escalated issue, the content of Cr in urban soil may be a neglected threat. Further pollution research in Mongolia should focus on street dust and drinking water pollution.

## Introduction

Mongolia is considered one of the most resource extraction-dependent countries globally^1^, and the mining industry has been a major contributor to its economy over recent decades^2,3^. The mining industry's origin is dated to 1924 when the first coal mine was opened in Nalaikh^2^. Its rapid development associated with the intensive release of mining licensing began in the 1990s after the transition to an open economy and is ongoing^4,5^. Simultaneously, the expansion of major official mining operations has been accompanied by artisanal and small-scale mining^6,7^. There are approximately 3,000 deposits and 50 different minerals explored and researched in Mongolia^1^, with the most economically important and mined commodities being black and brown coal, fluorite, Au, Cu, Ag, Mo, Pb and Zn^8,9^.

However, mining development and related economic growth have accelerated land degradation^10^. According to UNDP^5^, 77% of Mongolian land is classified as degraded or desertified. While mining-related environmental pollution in Mongolia has been intensively studied in recent years^7^, this is still insufficient because of the country's size and the number of mining areas. In the semi-arid to arid conditions of Mongolia^11^ with limited water resources^12^, water pollution poses a critical risk^3,4,12–16^. Insufficient scientific attention is paid to soil pollution, although mining of most minerals can be associated with soil contamination with metal(loid)s. Furthermore, there is no effective management system to assess the environmental health of mining sites^9^.

In recent decades, mining areas in Mongolia have been urbanised by originally nomadic inhabitants^12^. The concentration of people in these areas may be risky due to daily exposure to potentially hazardous substances in the environment released from mining operations and stored mining waste materials. Lkhasuren et al.^2^ stated that the high levels of dust associated with coal and gold mining are behind the high and growing number of lung diseases in Mongolia. Suvd et al.^6^ found typical chronic Hg intoxication symptoms in artisanal miners from gold mining areas in two sums. Recently, Surenbaatar et al.^9^ identified different patterns of mining's impact on children's health in areas of southern Mongolia. However, the results generally indicated the local effect of mining on the content of Pb in blood, As in urine and Hg in blood and hair.

The most resonant issue in urban soil pollution studies in Mongolia is coal combustion. Coal combustion can lead to a significant release of metal(loid)s into the environment and is ranked among the primary sources of contamination in the capital of Ulaanbaatar^17–20^, other cities^21^ and environments^22^. Other typical and frequently mentioned sources of contamination in Mongolia include transport and various industries^20,23^. However, the studies carried out so far on urban soil pollution with metal(loid)s in Mongolia strictly focus on the three largest cities (Ulaanbaatar, Darkhan, Erdenet), of which only Erdenet is directly linked to mining.

The pollution-related health risk may not only concern the immediate vicinity of mines. Due to aeolian dispersion and water erosion, the most common means of pollutants transport in arid and semi-arid environments^24^, metal(loid)s can be deposited over considerable distances. For example, mining activities in Mongolia are associated with the contamination of Lake Baikal in the Russian Federation^13,14^. Another problem may be traditional livestock grazing^4,11^. The accumulation of metal(loid)s in plants growing on polluted soils that livestock graze can endanger the vitality of the animals and the quality of food produced, leading to another human health risk^24,25^.

Given that the impacts of mining on soil pollution are serious and pose high health risks to the public in neighbouring, similarly highly mining-dependent China^26^, it can be assumed that mining also poses serious environmental and health risks in Mongolia. The absence of relevant studies on this issue points to the need for extensive research. The aims of this study are to: (1) assess the pollution of urban soils with metals in the key coal mining towns of Baganuur, Nalaikh and Sharyn Gol; (2) summarise and evaluate studies on soil pollution with metal(loid)s in Mongolia; and (3) assess the human health risks posed by soil pollution in Mongolia.

## Materials and methods

### Study area

Mongolia is characterised by extreme semi-arid to arid climate conditions, with an average annual rainfall reaching about 400 mm. Average annual temperatures range from approximately − 50 °C to 40 °C^27^. Strong winds that can easily enhance dust particles dispersion from mining areas significantly influence Mongolian steppe and desert areas. Wind erosion affects up to 90% of the country to some extent. The major soil types are Kastanozem soils, Cambisols and Chernozems^28^.

**Figure 1:**
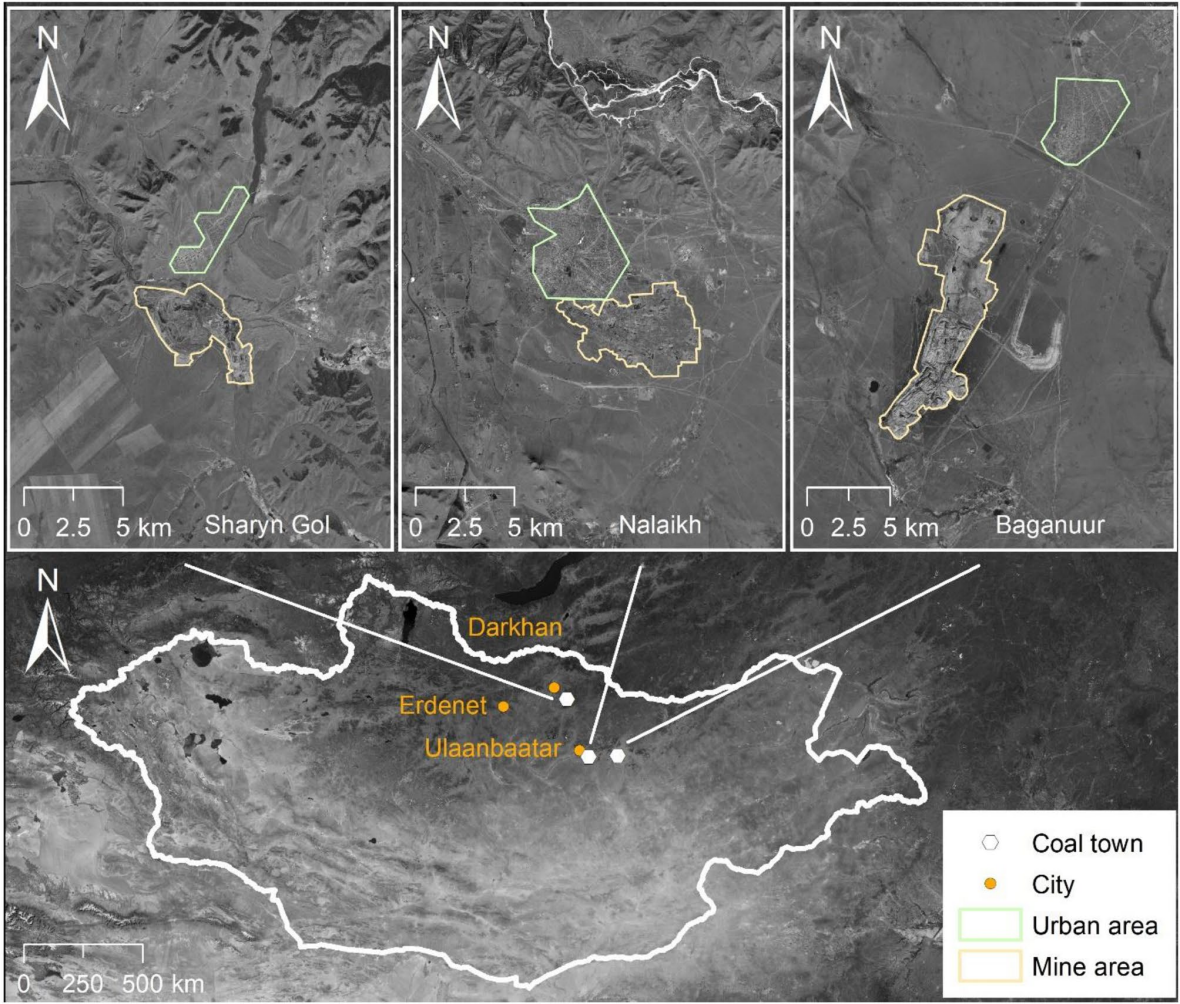
Location of the studied towns and three largest cities in the context of Mongolia. ArcGIS Desktop 10.8.1 software (Esri, CA, USA; https://www.esri.com/en-us/arcgis/products/arcgis-desktop) was used to create the map. The layers of satellite imagery were generated using Esri World Imagery basemap.

For this study, three key coal mining towns were sampled: Baganuur, Nalaikh and Sharyn Gol (also referred to as Sharyngol or Shariin Gol) (Fig. 1). These towns are or were main coal suppliers to the three largest Mongolian cities: Ulaanbaatar, Erdenet and Darkhan^17,29,30^. While official mining in Nalaikh has already ended^22^, small-scale illegal mining continues^7^. The open-pit (Baganuur, Sharyn Gol) or underground (Nalaikh) coal mines are to the south of the towns. The quality of mined coal varies between lignite and sub-bituminous^31,32^.

Furthermore, the available studies (up to 2021) were reviewed to evaluate the status of metal(loid) soil pollution in Mongolia.

### Soil sampling, preparation and analysis

Topsoil (0–10 cm) samples composited from three sub-samples from approximately 1 m^2^ were taken in each town according to a regular hexagonal sampling network. Forty-four samples were taken from Sharyn Gol, 50 from Nalaikh and 48 from Baganuur: 142 samples in total. The samples were air-dried at room temperature and sieved through a 2.0 mm nylon sieve following the standard ČSN ISO 11464.

The decomposition of the samples was performed in two replications according to ČSN EN 16174 by microwave extraction with aqua regia at the microwave extractor ETHOS ONE (Milestone, Italy). Half gram of soil sample was weighed to Teflon tubes, and 2 ml of Suprapur nitric acid (65%) and 6 ml of hydrochloric acid (37%) in ACS quality (Sigma Aldrich) were added. Extraction was performed at 175 °C for 20 min. Total metal (Cd, Cu, Pb and Zn) contents in soil samples were determined by flame atomic absorption spectrometry using Varian SpectrAA-30 (Varian, Australia). Air-acetylene flame atomisation (gas flow 13.5 L/min and 2.0 L/min) and an ultrasensitive hollow cathode lamp for Cd, Cu, Pb and Zn (Agilent Technologies, USA) were used. Cd, Cu, Pb and Zn were measured at 228.8 nm, 324.7 nm, 217.0 nm and 213.9 nm, respectively. For calibration, standard solutions of Cd, Cu, Pb and Zn (1 g/L) (Merck) and MilliQ water (Millipore, USA) were used. Certified reference material METRANAL 31 (light sandy soil; Analytika, Czech Republic) was used for quality control.

Based on the analysis of these basic metal pollutants, additional As, Cr, Hg and Ni content measurements were performed in several samples to verify the possibility of severe town-scale pollution with these metal(loid)s. The test samples were selected with regard to their increased content of Cd, Cu, Pb or Zn and diverse spatial distribution; up to fifteen samples were analysed in total. Contents of As, Cr and Ni in the soil samples were analysed using inductively coupled plasma mass spectrometer Agilent 7900 (Agilent, USA) with the SPS 4 autosampler (Agilent, USA) after microwave extraction with aqua regia at the microwave extractor ETHOS ONE (Milestone, Italy). Half gram of soil sample was weighed to Teflon tubes, and 3 ml of nitric acid (67–69%) and 9 ml of hydrochloric acid (36%) (both ANALPURE; Analytika, Czech Republic) were added. Extraction was performed at 200 °C for 15 min. For total Hg content analysis, a single-purpose atomic absorption spectrophotometer AMA-254 (Advanced Mercury Analyser; Altec, Czech Republic) was used without the necessity of further sample pre-treatment or pre-concentration. The quality control was the same as in the previous measurements. The median values of As, Cr, Hg and Ni in test samples were 10.9, 31.3, 0.03 and 17.4 mg/kg, respectively. Due to their prevailing contents being below the limits of the soil pollution standard^33^, they were not analysed in the remaining samples with the expectation of a low probability of pollution risk at the town scale.

### Soil pollution indicators and indices

The content of the metal(loid)s in soils was assessed in compliance with the permissible value of the Mongolian standard on soil quality (MNS 5850:2019)^34^ and the internationally recognised target and intervention values of the Dutch Soil Guidelines (DSG)^33^. Contamination/pollution indicators (indices) calculate the level of environmental contamination/pollution based mainly on local background values. However, the level of pollution determined in this way only weakly indicates the severity of the threat. Therefore, the adapted Integrated Nemerow Pollution Index (IPI_N_)^35^ with the Mongolian standard values (3, 100, 100 and 300 mg/kg for Cd, Cu, Pb and Zn, respectively) considered was chosen:

$${\text{PI}}_{{\text{i}}} = \frac{{{\text{C}}_{{\text{i}}} }}{{{\text{T}}_{{\text{i}}} }}$$

$${\text{IPI}}_{{\text{N}}} = \left[ {\left( {{\text{PI}}^{2}_{{{\text{avg}}}} { } + {\text{ PI}}^{2}_{{{\text{max}}}} } \right)/2} \right]^{1/2}$$

PI_i_ is the pollution index for a metal(loid), C_i_ is the content of the metal(loid), T_i_ is the permissible content of the soil pollutant given by MNS 5850:2019^34^, PI_avg_ is the mean value of all the PI_i_ of the metal(loid)s, and PI_max_ is the maximum PI_i_ value of the metal(loid)s. IPI_N_ classes are as follows: ≤ 0.7: safe; 0.7–1: precaution; 1–2: slight pollution; 2–3: moderate pollution; ≥ 3: heavy pollution.

### Human health risk assessment

The Hazard Index (HI) and Carcinogenic Risk (CR) were calculated to assess the potential health risk posed by metal(loid)s in Mongolian soils. The HI and CR calculations are based on average daily dose (ADD), reference dose (RfD), and slope factor (SF) values of metal(loid)s. Human exposure to metal(loid)s in soils (C_soil_) arises through ingestion (ADD_ing_), dermal contact (ADD_derm_) and inhalation. However, because ingestion and dermal contact pose the greatest health risks^35^, attention was paid only to these two pathways. An explanation of the input parameters and the values used for the calculations are summarised in Tables 1 and 2. To refine the authenticity of the calculation considering different physical features, national values (adult BW, LT) and values used in neighbouring China (children BW, SA) were used instead of the conventional ones. The following equations were used for the intake estimations via each exposure pathway^36^:

$${\text{ADD}}_{{{\text{ing}}}} { } = \frac{{{\text{C}}_{{{\text{soil}}}} \times {\text{ IngR }} \times {\text{ EF }} \times {\text{ ED}}}}{{{\text{BW }} \times {\text{ AT}}}}{ } \times { }10^{ - 6}$$

$${\text{ADD}}_{{{\text{derm}}}} = { }\frac{{{\text{C}}_{{{\text{soil}}}} \times {\text{ SA }} \times {\text{ AF }} \times {\text{ ABS }} \times {\text{ EF }} \times {\text{ ED}}}}{{{\text{BW }} \times {\text{ AT}}}}{ } \times { }10^{ - 6}$$

**Table 1:** Calculation parameters.

Factor	Description	Unit	Adult	Children	References
IngR	Ingestion rate of soil	mg/day	100	200	USEPA^38^
EF	Exposure frequency	days/year	350	350	MEP^39^
ED	Exposure duration	years	24	6	USEPA^38^
BW	Bodyweight	kg	65.0^a^	15.9^a^	MEP^39^
AT	Average time	days	8760^b^	2190^b^	Pecina et al.^35^
SA	Exposed skin surface area	cm^2^	4350	1600	Pecina et al.^35^
AF	Skin adherence factor for soil	mg/cm^2^	0.07	0.20	Pecina et al.^35^

^a^BW was calculated as the average of the adult Mongolian weights reported in WHO^40^, Otgontuya et al.^41^, and WHO^42^ studies. The Mongolian children's weight values were not found; therefore, the values from neighbouring China^39^ were used.

^b^For carcinogenic effects^43^: LT (lifetime) × 365. In Mongolia^44^, LT = 70. For carcinogenic effects, AT = 25 550.

**Table 2:** The relative toxicity values used (based on the literature summary in Pecina et al.^36^).

Metal	RfD_ing_	RfD_derm_	ABS^a^	SF_ing_
Cd	1.00E-03	1.00E-05	0.001	1.50E+01
Cu	4.00E-02	1.20E-02	0.001	
Pb	3.50E-03	5.25E-04	0.001	8.50E−03
Zn	3.00E-01	6.00E-02	0.001	

^a^Dermal absorption factor.

Total non-carcinogenic health risk, HI, was determined as a sum of calculated individual hazard quotients (HQ)^30,37^:

$${\text{HI}} = \sum {\text{ HQ}}_{i} { } = { }\sum \frac{{{\text{ADDi}}}}{{{\text{RfDi}}}}$$

HI values > 1 indicate the probability of non-carcinogenic adverse health effects.

The carcinogenic risk was calculated for considered carcinogenic metals (Cd and Pb) as follows^35^:

$${\text{CR}} = \sum {\text{ ADD}}_{{\text{i}}} { } \times {\text{ SF}}_{{\text{i}}}$$

CR values ≤ 10^–6^ indicate virtual safety, whereas values ≥ 10^–4^ indicate an unacceptable risk. The acceptable risk range for regulatory purposes is 10^–6^ to 10^–4^.

### Spatial data analysis

Statistica 12® was used for statistical analyses of the dataset. Differences in the soil contents of metals between the towns were tested. Data normality was investigated using the Shapiro–Wilk normality test (*P* > 0.01). One-way ANOVA was used for normal series; if at least one of the series did not pass the normality test, a nonparametric Kruskal–Wallis ANOVA was used. Spatial data of the pre-processing and geostatistical analysis were conducted in SW ESRI ArcGIS Desktop 10.8 using the Geostatistical Analyst extension. All output rasters of the interpolated values were calculated with a spatial resolution of 10 m. The geostatistical kriging method was selected for spatial data interpolation, specifically, Empirical Bayesian Kriging where the mean prediction errors are more accurate for small data sets compared to other kriging methods. Spatial data transformation (with any base function) was not applied. The thin Plate Spline semivariogram model was chosen by testing several different models of semivariogram. Setting the semivariogram has always been simulated for 100 models, and the results for the map outputs were checked by cross-validation. A smooth circular interpolation option with a radius of 500 m and a smoothing factor of 0.5 was selected in search neighbourhood parameters to control the output.

## Results and discussion

### Soil pollution in the coal mining towns

**Figure 2:**
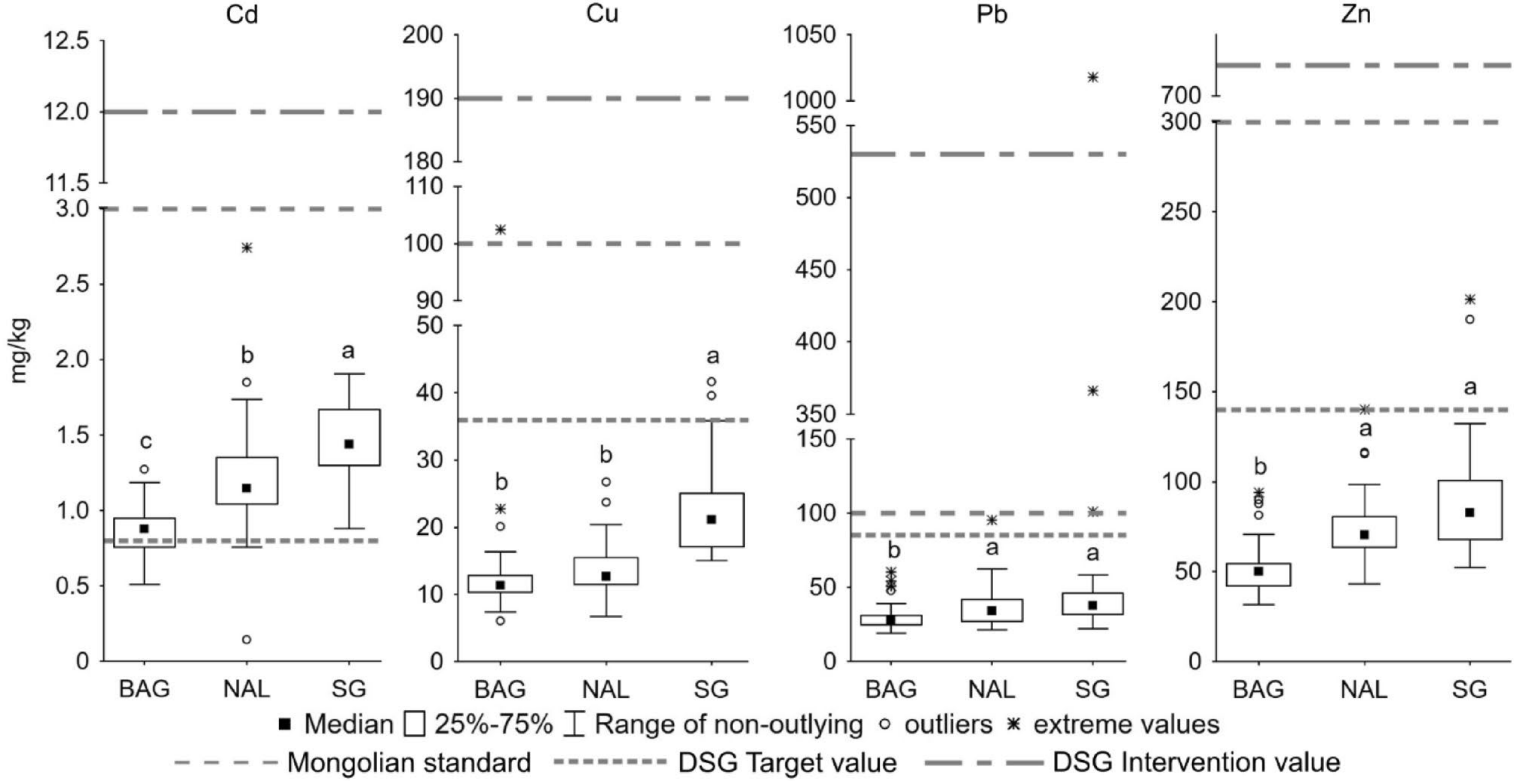
Metal contents (mg/kg) in the urban soils of the coal mining towns.

The average contents of metals did not exceed the Mongolian standard^34^ in any of the studied towns (Fig. 2). The standard was rarely exceeded, once in Cu (Baganuur) and three times in Pb (Sharyn Gol). Cd exceeding the target value^33^ in Baganuur, Nalaikh and Sharyn Gol (Fig. 2) indicates contamination with this element. Pb exceeded the intervention value, suggesting severe pollution, only once (Sharyn Gol). IPI_N_ assessment classified all the towns as safe according to average IPI_N_ values (Table S1). Therefore, despite the ongoing open-pit mining (Baganuur, Sharyn Gol) or historical underground mining (Nalaikh) of coal enriched with chalcophile elements^7,17,23,29^, the results suggest minor pollution with Cd, Cu, Pb and Zn.

**Figure 3:**
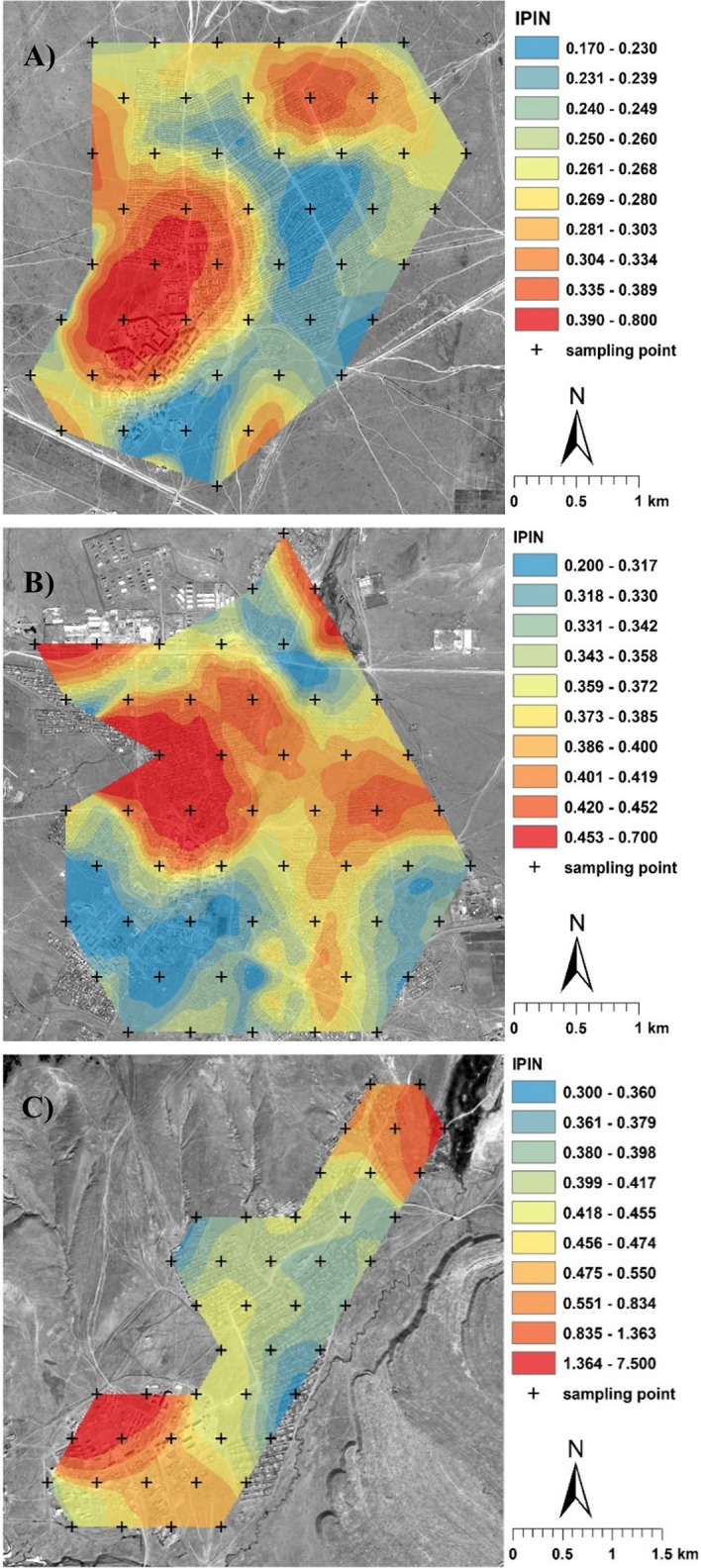
Integrated Nemerow Pollution Index (IPI_N_) assessment-based pollution distribution in the coal mining towns: (**A**) Baganuur, (**B**) Nalaikh, and (**C**) Sharyn Gol. ArcGIS Desktop 10.8 software (Esri, CA, USA; https://www.esri.com/en-us/arcgis/products/arcgis-desktop) was used to create the map. The layers of satellite imagery were generated using Esri World Imagery basemap.

Dependence of the soil contamination to pollution distribution on the mine's proximity is rather indirect or none (Fig. 3), with predominantly other determining factors. Similarly, Nottebaum et al.^7^ did not find an immediate or even dominating impact of mining on soil contamination with As in Nalaikh. Thus, mining does not cause pollution there (Fig. 2, Table S1) and can hardly even be associated with local contamination tentatively indicated by slightly increased IPI_N_ values (Fig. 3).

With Baganuur and Sharyn Gol, the signs of contamination to pollution are mainly in the original town centre with paved roads and multi-storey buildings (Fig. 3). The assumption is that there has been more intensive traffic that is associated with higher emissions of metals. Furthermore, the general coverage of the soil with impermeable surfaces (e.g. buildings, parking lots) could increase the occurrence of pollution hotspots in limited places that have long allowed the retention and accumulation of contaminating particles. However, the situation was the opposite in Nalaikh, with higher IPI_N_ values in ger areas.

The division of residential areas into areas with multi-storey buildings and gers is such a unique Mongolian phenomenon that it is addressed in other studies. Coal is the primary energy source in ger areas, and burning-generated ash is becoming one of the primary sources of in situ soil pollution due to improper disposal^45,46^. Kasimov et al.^47^ stated that the soils of the districts with multi-storey buildings are more polluted than the ger districts. Chonokhuu et al.^37^ found different pollution patterns in this regard, as in Ulaanbaatar and Erdenet, ger area > apartment area, while in Darkhan, apartment area > ger area; and explain them by different socio-economic features and ageing of cities. However, Bilguun et al.^20^ found the opposite pollution order in Ulaanbaatar as downtown > suburban settlement > ger area. These results suggest that there may be considerable variability or inappropriate sampling methodology even within one city. An additional verifying statistical comparison was not applicable for the studied towns due to the limited number of samples from the areas with multi-storey buildings or their borderline characters.

The reason for the different patterns in urban design-moderated pollution distribution in Nalaikh compared to Baganuur and Sharyn Gol may be the utilisation of low-quality coal with a higher content of metals. Due to frequent small-scale illegal mining in Nalaikh^7^, such coal without quality control may be often utilised in ger areas, dispersing more contaminated ash and increasing pollution.

Only an increase in IPI_N_ values in the north-eastern part of Sharyn Gol (Fig. 3C) can be directly linked to mining. Generally, the higher IPI_N_ values in this town (Table S1) may be related to the mining waste heaps deposited near it in the east. The contents of Cd, Cu, Pb and Zn are mostly increased there, about 1.91, 40.5, 58.4 and 104 mg/kg, respectively (own unpublished data). Due to the absence of vegetation cover or other protection against aeolian transport, dust particles containing metal(loid)s can be easily introduced into the town. Hills form the northern and western borders of the town, and a mine and other heaps form the southern one. The town's location in this valley increases the risk of sedimentation and accumulation of contaminants.

### Urban soil pollution in Mongolia

So far, studies performed on urban soil contamination with metal(loid)s in Mongolia have focused strictly on the three largest cities (Table 3). In addition, the limit of these studies is a low number of samples, and the limited sampling design only focused on some parts of the city or a specific phenomenon (e.g. roadside soils, ger areas). Therefore, they do not provide a comprehensive view of the actual pollution situation in the cities. The quality of some may also be questioned due to insufficient or unreliable descriptions of results or methodologies. However, their results suggest that soil pollution in Mongolia is less severe than expected.

**Table 3:** Summary of studies on contamination of Mongolian urban soils with metal(loid)s (average values in mg/kg).

City/Town	n	As	Cd	Cr	Cu	Hg	Ni	Pb	Zn	References
Baganuur	48		0.87		13.6			29.6	51.7	This study
Darkhan	25*			704**	50.8	0.06	32	29.2	63.6	Kosheleva et al.^21^
	21	3.33		31.9			19.5	20.9	67.3	Chonokhuu et al.^37^
	18*^1^	5.22	0.23	32.1	18.5		15.2	29.9	82.3	Timofeev et al.^30^
Erdenet	18*			113	**450**	0.10	35	24.7	62.2	Kosheleva et al.^21^
	30	12.8		65.7			29.3	18.1	155	Chonokhuu et al.^37^
Nalaikh	50		1.21		13.8			36.4	73.7	This study
Sharyn Gol	44		1.46		22.1			69.0	87.6	This study
Ulaanbaatar	22	14	0.8	20.3	35.9		18.7	63.9	159	Batjargal et al.^17^
	23*			72.6	56.5	0.19	30.6	59.4	105	Kosheleva et al.^21^
	90	10.8	0.25	32.7	22.4		17.0	45.9	120	Kasimov et al.^47^
	29					0.09				Chung and Chon^19^
	10							39.1		Tserenpil et al.^48^
	x***	10.3		**685**				33.0	113	Naidansuren et al.^49^
	27	28.0		16.6			21.3	43.1	106	Chonokhuu et al.^37^
	42	22.9	0.2	29.0	28.9			34.5	136	Battsengel et al.^45^
	22	9.41	0.25	30.0	20.8			45.1	116	Bilguun et al.^20^
	28	16.4		**1987**	53.5		13.6	33.2	111	Oyunbat et al.^50^
Mongolian standard		20	3	150	100	2	150	100	300	MNS 5850:2019^34^
DSG target value		29	0.8	100	36	0.3	35	85	140	VROM^33^
DSG intervention value		55	12	380	190	10	210	530	720	VROM^33^

*n* number of samples, *DSG* Dutch Soil Guidelines; studies exceeding all the standards are bold.

*Data selected from the study's category with the most samples.

**Possible incorrect value inconsistent with the text of the article.

***XRF readings on 340 points.

Cadmium is one of the most important contaminants in urban soils^35^; however, its contents were atypically low (≤ 0.25 mg/kg) in most studies or have not been studied (Table 3). The exceptions are the coal mining towns, which exceeded the DSG target value. Arsenic in the urban soils (Table 3), originating mainly from coal combustion^20,47,51^, regularly (80%) exceeded the former Mongolian limit (6 mg/kg; MNS 5850:2008), which was usually used in the studies concerned. However, the value given by that standard can generally be considered extremely low, even compared to background values. In the context of DSG target value and current Mongolian standard^34^, the urban soils content of As is rather low. In addition, Nottebaum et al.^7^, partly studying urban soils of Nalaikh, stated that As does not pose a ubiquitous risk there. Extreme Cr contents (Table 3) exceeding DSG intervention value in Ulaanbaatar (reaching 1987 mg/kg) and possibly in Darkhan (704 mg/kg) are associated with local pollution by the leather processing industry^21,49,50^. Given the importance of the leather processing industry using Cr-based technology in traditionally pastoral Mongolia^52^, similarly significant pollution with Cr can be expected elsewhere.

Increased Cu content (450 mg/kg) compared to other cities and standards (Table 3) was found only in Erdenet, where Cu-Mo mining occurs. Timofeev et al.^51^ also found soil contamination with Cu in the Erdenet area. The Hg contents (Table 3) commonly associated with coal combustion are surprisingly low^19^. This finding may be misrepresented by the low number of studies addressing this element. However, Chung and Chon^19^ explain that Ulaanbaatar's Hg contamination is lower than in other cities with analogous circumstances because of the lower Hg content in the coal used and the spatial and temporal trends in coal usage. The contents of Ni and Pb (Table 3) are also low and balanced between cities, which indicates their low anthropogenic emissions or similar contamination patterns without risk expectations. The increased Zn values in Ulaanbaatar compared to other cities (Table 3) may be related to: (I) different natural backgrounds, (II) long-range transport of Zn emissions from the heavy industry area in China potentially affecting Ulaanbaatar^18^ or (III) higher local emissions of Zn. However, we can relatively reject hypotheses I and II, given the possible background values (Table 4). Therefore, an important local source of Zn is expected. This may be related to heavy traffic^53^ and various industries because these sources of Zn are expected to be important in Ulaanbaatar^20^. However, Zn contents are also low compared to the standards (Table 3).

**Table 4:**
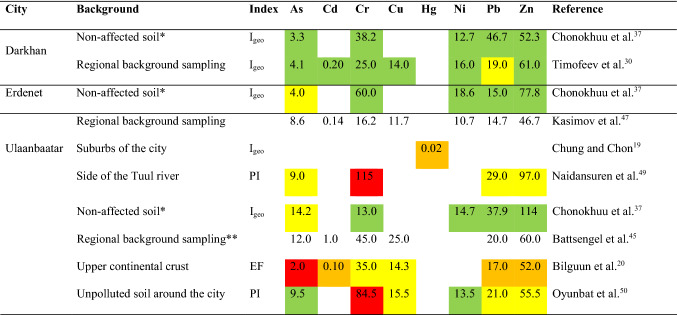
Background values (mg/kg) used in the studies on urban soil pollution in Mongolia together with generalised level of contamination/pollution calculated based on these background values (where clearly indicated).

Colours signals "contamination/pollution" level found/indicated in the respective study: green = none, yellow = low/minor, orange = moderate, red = high/heavy; I_geo_ = geoaccumulation index, *EF* enrichment factor, *PI* pollution index.

*Based on the references (local studies) and the composition of the continental crust.

**Cited from the local source studying background concentration of microelements in Ulaanbaatar's regional natural surface soil.

Due to the different calculation methods, the pollution level mentioned in the summarised studies is not comparable. Despite the oft-cited moderate or high contamination or pollution (the ambiguity is mainly based on incorrectly used terminology) by at least one element (Table 4), comparisons with the standards (Table 3) suggest considerably low contamination and no pollution. A notable exception is the study of Chonokhuu et al.^37^, reporting the predominant absence of contamination in the three largest Mongolian cities. However, there is no discussion of other studies' results concerning these cities or a better explanation of the results, reducing the credibility of the conclusions.

An overall weakness in the soil contamination assessment is the lack of knowledge of background values or their usage inconsistency, which can distort the actual situation significantly. Studies assessing local contamination often used general standardised values, such as Kosheleva et al.^21^, Chonokhuu et al.^37^ and Bilguun et al.^20^. Using local background values is less common, and the values for the same cities vary considerably in different studies (Table 4). As a result, for example, Cr values ranging from 13.0 to 115 mg/kg are used as a background in the Ulaanbaatar. The disagreement emphasises the need for a better methodological basis for sampling. This finding also indicated that the reality of contamination of Mongolian urban soils might differ from what the studies suggest, as there is no credible background that the authors agree with at the local level.

### Soil pollution in Mongolian mining areas

Studies on soil pollution related to mining in Mongolia can be divided into two groups: I) accompanying soil assessment in the study of water contamination and II) assessment of mining areas partly including urban units. The studies concern three basic mineral resources: Au (Boroo, Zaamar) Cu (Erdenet) and coal (Baganuur, Nalaikh).

- *Au* The results of Inam et al.^54^ suggest that, except for As (3.4–261 mg/kg), soil metal(loid) contents are within the acceptable levels of the Mongolian standard^34^ in the vicinity of the Boroo gold mine. However, the authors pointed out that the waste material may be a source of future contamination. Later, Oyuntsetseg et al.^55^ found Cu, As and Pb enrichment in soils at the Boroo small-scale mining area that exceeded the standard in As (167 mg/kg) and Cu (109 mg/kg) in the gold washing location. In Zaamar Goldfield, Jarsjö et al.^56^ found elevated soil contents of As (13 mg/kg), Ni (28 mg/kg), Cu (25 mg/kg) and Cr (69 mg/kg) compared to non-local backgrounds; however, no metal(loid) exceeded the current standard. Thus, only As can pose a potential threat in limited and extreme cases of gold mining operations.
- *Cu* Most attention has been focused on Cu-Mo mining in Erdenet. Battogtokh et al.^57^ stated that the soils in the Erdenet mining area are highly contaminated with Cu. While Timofeev et al.^51^ designated the studied soils as urban, we assigned the overall study conclusions to this chapter due to the predominance of out-of-city sampling. The authors found contamination and potential environmental risk related to Cu and As exceeding the former Mongolian standard. Yondonjamts et al.^58^ achieved the same conclusions. Dust production by grinding and transportation associated with the mine operation and wind erosion of technogenic sands of the tailings were highlighted as important factors for contamination of the mining area^51,57,59^.
- *Coal* Park et al.^60^ expect the risk of As poisoning due to above-standard (former) As contents (5.57–14.2 mg/kg) in the soil dust from the Baganuur mine; however, they draw conclusions only from five samples and still low As contents in the context of other standards (Table 3). Nottebaum et al.^7^ found As contents in Kastanozem topsoil and fluvial-alluvial sediments of the Nalaikh area ranging from 4.6 to 16 mg/kg, and consider them low and not a serious threat to the environmental compartments. Differences in conclusions, despite similar values, highlight the inconsistency in the evaluation of results reported in Mongolian studies.

### Risk assessment and future perspectives

Although many of the mentioned studies highlighted the issue of soil pollution with As in particular, its content is predominantly low by other international standards (Table 3). Assumedly, the threat of As in the Mongolian soils is considerably artificially escalated in the context of a low value of the former Mongolian standard.

Given that soil contents of metal(loid)s in Mongolia are predominantly low (Table 3, Chapter 3.3), a low health risk may be expected. Chonokhuu et al.^37^ confirmed this assumption, not finding serious health threats based on potential health risk assessments in Ulaanbaatar, Darkhan or Erdenet. Conversely, Battsengel et al.^45^ emphasised possible health risks in Ulaanbaatar. However, the description of the results in both studies suggests a partial misunderstanding of the calculation, as the authors present the generally applicable facts arising from the calculation methodology as unique results. In addition, errors can be found in both cases. The conclusions of Battsengel et al.^45^ can, therefore, be considered incorrect.

According to a potential human health risk assessment (Table 5), there are no non-carcinogenic or carcinogenic risks in the coal mining towns posed by the soil contents of the metals. Only CR values of Cd indicated a possible threat as they fell into the acceptable total risk category for regulatory purposes.

**Table 5:** Average values of human health risk assessment via potential non-carcinogenic (HI) and carcinogenic risk (CR) following different exposure pathways in the coal mining towns.

		Cd				Cu		
		HQingest	HQdermal	HI	CR	HQingest	HQdermal	HI
Baganuur	Adult	1.28E−03	3.90E−04	1.67E−03	6.59E−06	5.03E−04	5.11E−06	5.09E−04
	Children	1.05E−02	1.68E−03	1.21E−02	1.35E−05	4.12E−03	2.20E−05	4.14E−03
Nalaikh	Adult	1.79E−03	5.44E−04	2.33E−03	9.19E−06	5.09E−04	5.16E−06	5.14E−04
	Children	1.46E−02	2.34E−03	1.69E−02	1.88E−05	4.16E−03	2.22E−05	4.18E−03
Sharyn Gol	Adult	2.15E−03	6.55E−04	2.81E−03	1.11E−05	8.16E−04	8.28E−06	8.24E−04
	Children	1.76E−02	2.82E−03	2.04E−02	2.26E−05	6.67E−03	3.56E−05	6.71E−03

		Pb				Zn		
		HQingest	HQdermal	HI	CR	HQingest	HQdermal	HI
Baganuur	Adult	1.25E−02	2.53E−04	1.27E−02	1.27E−07	2.54E−04	3.87E−06	2.58E−04
	Children	1.02E−01	1.09E−03	1.03E−01	2.60E−07	2.08E−03	1.66E−05	2.10E−03
Nalaikh	Adult	1.53E−02	3.12E−04	1.57E−02	1.57E−07	3.62E−04	5.52E−06	3.68E−04
	Children	1.25E−01	1.34E−03	1.27E−01	3.20E−07	2.96E−03	2.37E−05	2.99E−03
Sharyn Gol	Adult	2.91E−02	5.91E−04	2.97E−02	2.97E−07	4.31E−04	6.56E−06	4.37E−04
	Children	2.38E−01	2.54E−03	2.40E−01	6.07E−07	3.52E−03	2.82E−05	3.55E−03

Low metal contents in Mongolian mining areas (Fig. 2, Table 3, Chapter 3.3) and the associated low health risks contrast sharply with other mining areas of the world^26,36^. The reason may be a short history of mining. While intensive mining and mineral processing has been taking place for several centuries in some countries, the mining industry is still a young sector in Mongolia^2^. Limited precipitation and low humidity typical for Mongolia and the sorption stability of elements in lignite and sub-bituminous coal likely reduce weathering of mining waste and release of metal(loid)s into the environment within such a short time. Furthermore, limited local ore processing followed by direct export could also contribute to the current favourable situation. The last important factor is probably the wind erosion and wind transport of dust particles far from the deposits. However, the positives of this phenomenon can be ambiguous as particle accumulation must occur somewhere, and the creation of pollution hotspots can be expected.

A significant threat posed by the aeolian dispersion of contaminated dust particles from mining waste heaps is indicated in Sharyn Gol (Fig. 3C) and mentioned as risky in other related studies^7,57,59^. Park et al.^60^ even stated that all waste soil samples from Baganuur appeared to have desertification potential. A possible solution for the dust risk decrease is phytostabilisation. Extreme conditions of mining waste, such as acidic pH, lack of nutrients, unsuitable substrate structure, and instability^61,62^, can be mitigated using available organic materials, such as wood chips or manure^24^. This topic should be addressed by the relevant authorities and further research.

Poor management of mining sites often leads to livestock grazing on sparse vegetation of heaps and drinking from tailings dams in Mongolia (own observation). The risk of livestock intoxication and unsuccessful phytostabilisation must be reduced by fencing and permanent monitoring.

## Conclusions

The results showed that the contents of Cd, Cu, Pb and Zn are low without significant pollution and health risk in the coal mining towns of Baganuur, Nalaikh and Sharyn Gol. Based on the available studies, the same conclusions can be drawn for other Mongolian cities. Only the Cr content associated with the leather industry can likely pose a significant threat and, therefore, warrants increased attention. The generally highlighted risk of As contamination, on the contrary, acts more like a virtual problem. Many soil contamination studies focused on Mongolia are based on the inappropriate methodology or an insufficient number of samples, reducing their results' quality and conclusions' relevance. Due to the potential for the long-term growth of the Mongolian mining sector, a precise metal(loid)s contamination assessment is highly needed. Given the results, future research on pollution in Mongolia should focus more on drinking water and street and mining dust pollution.

## Supplementary Information

Supplementary Information.

## Data Availability

The datasets generated during and/or analysed during the current study are available from the corresponding author on reasonable request.
