# Supplementary material for: The impacts of mining on soil pollution with metal(loid)s in resource-rich Mongolia
Source: Sci Rep. 2023 Feb 16;13:2763. doi: 10.1038/s41598-023-29370-w (PMC9935523; doi:10.1038/s41598-023-29370-w)
Supplement: Supplementary file 1 — Supplementary Information. [file 41598_2023_29370_MOESM1_ESM.docx]

# **Supplementary Material**

# **The impacts of mining on soil pollution with metal(loid)s in resource-rich Mongolia**

Václav Pecina ^a, *^, David Juřička ^b^, Josef Hedbávný ^c^, Martin Klimánek ^d^, Jindřich Kynický ^e^, Martin Brtnický ^a,f^, Renata Komendová ^a^

*^a^ Institute of Chemistry and Technology of Environmental Protection, Faculty of Chemistry, Brno University of Technology, Purkyňova 118, 61200 Brno, Czech Republic*

*^b^ Department of Geology and Soil Science, Faculty of Forestry and Wood Technology, Mendel University in Brno, Zemědělská 3, 613 00 Brno, Czech Republic*

*^c^ Department of Chemistry and Biochemistry, Faculty of Agrisciences, Mendel University in Brno, Zemědělská 1, 613 00, Brno, Czech Republic*

*^d^ Department of Forest Management and Applied Geoinformatics, Faculty of Forestry and Wood Technology, Mendel University in Brno, Zemědělská 3, 61300 Brno, Czech Republic*

*^e^ BIC Brno, Technology Innovation Transfer Chamber, Purkyňova 125, 612 00, Brno, Czech Republic*

*^f^ Department of Agrochemistry, Soil Science, Microbiology and Plant Nutrition, Faculty of AgriSciences, Mendel University in Brno, Zemědělská 1, 613 00 Brno, Czech Republic.*

** Corresponding author, e-mail xcpecina@fch.vut.cz; ORCID 0000-0003-4274-5142*

**Table S1**. Integrated Nemerow Pollution Index (IPI_N_) assessment.

|  |  | **IPI_N_** |
| --- | --- | --- |
| **Baganuur** | Average | 0.28 |
|  | S.D. | 0.10 |
|  | Min. | 0.17 |
|  | Median | 0.26 |
|  | Max. | 0.80 |
| **Nalaikh** | Average | 0.38 |
|  | S.D. | 0.09 |
|  | Min. | 0.22 |
|  | Median | 0.36 |
|  | Max. | 0.74 |
| **Sharyn Gol** | Average | 0.65 |
|  | S.D. | 1.10 |
|  | Min. | 0.26 |
|  | Median | 0.43 |
|  | Max. | 7.49 |
